# Originality of Method

**Published:** 1889-09-07

**Authors:** 


### The Hospital

A Weekly Institutional Journal of

Science, Medicine, Nursing, and Philanthropy.

*For Hospitals, Asylums, Medical Practitioners, Students, Nurses, Families, Parishes, Congregations, and the Charitable Public.*

Vol. VI. No. 154.] [September 7, 1889.

### Originality of Method.

"There is an originality of method," says Dr. Hughlings Jackson; "the man who puts two old facts in new and more realistic order deserves praise as certainly as does the man who discovers a new one. Gaskell has found out much that is new in his recent well-known researches on the nervous system; but I imagine that the greatest merit of his work is that he has made fruitful generalisations; and has set many things, long ill-assorted, in realistic order." This is the same as saying that he who teaches geography with skill and success is a man of merit as decidedly as is he who discovers a new island or ascertains the peculiarities of a race of hitherto undescribed savages. The teacher may in fact not only be as meritorious as the discoverer, but much more so. For after all, what is it which makes most men discoverers? Is it not usually opportunity? And if the discoverer have opportunity without teaching power his work is seldom of much service to mankind. Darwin had rare opportunities for the acquisition of knowledge. He had also an unusual skill and charm of style in placing his knowledge before others. The conjunction of vast and varied knowledge with teaching power made him Darwin. Huxley, happily for the world, began his career as an explorer. His passion for concrete facts made him also a practical worker. The same passion has constantly impelled him to try to make the world see facts as he has seen them. His originality consists in a strong desire to see things as they are, and an equally strong determination to make other people see them in the same way. As mere discoverers both these men might have been of little moment in the world, but as discoverers full of enthusiasm and endowed with teaching power they have not only filled the world with their fame, but made it their debtor for all time.

For want of an adequate conception of the value of teaching power, the medical profession, as well as some others, is much retarded in its onward progress. Every young medical man of more than average mental capacity and industry sets himself, as the shortest cut to fame, on the pathway of discovery. Now a great many important things in anatomy, physiology, and practical medicine have already been discovered; and the unexplored fields are becoming daily narrower and more limited in extent. The result is that in these times many so-called discoveries are of little consequence, adding next to nothing to knowledge, and nothing at all to practical progress. But inasmuch as they are new discoveries, they are spoken of as if they must necessarily be great in themselves and the proof of greatness in those who have brought them to light. There is no attempt made to value them or to measure the general worth of their discoverers. Both are, on the strength of what may be nothing more than an accident, alike placed on an elevated pinnacle of honour for all men to wonder at. This is a very great injury not only to the discoverers themselves, but also to the cause of scientific advancement.

The medical profession as a whole is not a well-taught and highly-disciplined body of men. The books that medical men have to read, and which are, so to speak, the bibles of their daily professional faith and practice, are, with few exceptions, long, windy, ill-digested, and illogically constructed masses of fact and tradition, which often leave their readers more hopelessly in the dark than before. There is at the present moment the most urgent necessity for what Dr. Hughlings Jackson has called "an originality of method." But no encouragement is offered to young men of teaching or expounding power to put their gifts to their natural use. All the honours are reserved for the discoverers, and therefore all men, teachers and others, try to become discoverers. Even teaching appointments themselves are not given to men because they can teach, but because they have had unusual opportunities or unusual good fortune in the fields of discovery—"original research," as it is called. No doubt new knowledge is badly needed; but will any one for a moment contend that the discovery of even so important a thing as a new anæsthetic would be of so much service to the medical profession as a whole, and through it to the world, as the intelligent comprehension by every practitioner of the vast stores of knowledge already accumulated, and the power of applying that knowledge in daily practice? As a matter of fact, large numbers of the rank and file of the profession are half a century behind the skilled leaders. Their methods are out of date; their knowledge is nothing. The reason of this, as we have said, is that all the highest talent is enticed into the fields of discovery, and the power of full and lucid exposition is neglected and despised.

We do not undervalue new knowledge; on the contrary, we see clearly and plainly the urgent necessity there is for a profounder acquaintance with diseased processes, and with the means at hand for preventing, arresting, or guiding them to a safe issue. But we contend that if the practical knowledge of these processes and of the means now available for successfully dealing with them were made the intelligent possession of the whole body of practitioners, new knowledge itself would be more rapidly acquired, whilst progress on every line would advance with tenfold speed. For what, after all, is the true and proper business of medicine? Is it the mere amassing of mountains of hard facts, or is it the ordering and arranging of amassed knowledge on such lines that it may be made most promptly and widely available for the needs and distresses of man? Is the recruiting sergeant who brings in the raw ploughman or navvy from the field more to be admired than his brother sergeant who teaches the goose-step; or is either of them to be compared for a moment with the bold and skilful general who marshals the whole army to victory? Physic at the present moment seems to be at that crude stage which gives all the honours to the recruiting sergeant, with the result that a really able general is looked for in vain.

Medicine can never advance within its own domain or be understood and valued at its proper worth by the world outside until it makes use of the methods of progress and exposition which literature puts into its hands. It is a science as well as an art. As a science it is by its own nature the property of mankind. No limited class has any right to the exclusive possession of any kind of natural knowledge whatever. As an art it must be taught, and no art rises beyond the general level of the teaching capacity of its teachers. Here and there exceptional genius may show exceptional results, but the average level of any practised art must be the average teaching level of those who ordinarily expound it. This is all as plain and self-evident as a mountain in the morning light to any man of ordinary capacity. If medicine is to hold its own and to win, as it is entitled to win, a much higher position than it now possesses in the world's esteem, it must reconsider both its judgments and its methods. Leaders must be chosen, not because they have been more or less successful recruiting sergeants, but because they possess wide culture and large experience of practical affairs. Teachers must be selected because they can teach, and they must be held in esteem and receive such rewards as the high value of their services renders just and equal. As well might we hope to have poetry without language, or oratory without speech, as that a practical science and art like medicine shall be understood by its followers or valued by the world without the light with which teaching genius can illuminate it on all sides. Give us discoverers by all means, but give us also those who can weigh and measure each discovery, and can make all men see both its exact value and its true place in that general scheme of things which we call "the world."

